# Flexoelectricity in Pyramid-Patterned Contact Areas of NOA/Ecoflex Triboelectric Nanogenerators

**DOI:** 10.3390/nano16140855

**Published:** 2026-07-11

**Authors:** Nursalim Akhmetzhanov, Dong-Joo Kang, Jong-Man Kim, Dong-Myeong Shin, Yoon-Hwae Hwang

**Affiliations:** 1 Department of Nano Fusion Technology & BK FOUR Nanoconvergence Technology Division, Pusan National University, Busan 46241, Republic of Korea; akhmetzhanov.nursalim@gmail.com (N.A.); jongkim@pusan.ac.kr (J.-M.K.); 2 School of Transdisciplinary Engineering, Pusan National University, Busan 46241, Republic of Korea; kdj9880@kakao.com; 3 Department of Mechanical Engineering, The University of Hong Kong, Pokfulam Road, Hong Kong, China; dmshin@hku.hk

**Keywords:** triboelectric nanogenerator, flexoelectricity, NOA-63, Ecoflex, pyramid microstructure, contact electrification, energy harvesting

## Abstract

This study investigates flexoelectricity in the pyramid- and truncated-pyramid-patterned contact interface of a NOA-63/Ecoflex (N/E) triboelectric nanogenerator (TENG) operating in contact–separation mode. Microscale pyramidal and truncated pyramidal arrays were fabricated using silicon molds and paired with an Ecoflex™ 00-10 elastomer substrate, and the structural integrity of the arrays was confirmed by scanning electron microscopy. Combining experimental results with established knowledge of soft-polymer indentation mechanics and hard-to-hard flexoelectric behavior, the surface charge density (σ) and flexoelectric coefficient (μ_flexo_) were determined to be 8.48 × 10^−6^ C/m^2^ and 2.95 × 10^−11^ C/m, respectively. These parameters were incorporated into a total charge equation to estimate charge output for both pyramidal and truncated pyramidal N/E TENG arrays under varying applied loads. The proposed model can adequately predict the charge output of pyramidal and truncated pyramidal N/E TENGs.

## 1. Introduction

Nanogenerators are compact energy-conversion devices capable of powering low-consumption electronics, making them central to the ongoing development of the Internet of Things (IoT). As IoT systems trend toward miniaturization and operational autonomy, flexible power sources have become a key technological priority. Although wind–solar hybrid systems serve many remote IoT nodes, no clear solution yet exists for flexible, body-worn electronics.

Flexible and stretchable electronics are critical enablers of wearable health monitors [1,2,3,4], curvilinear prosthetic interfaces [5], and conformal antennas [6]. Wearable devices that provide continuous physiological monitoring can substantially improve quality of life for those with individual chronic medical conditions [1,2,3,4]. Antennas, which are frequently subjected to mechanical bending, would benefit greatly from self-powered operation enabled by flexible nanogenerators. Among all flexible nanogenerator architectures, triboelectric nanogenerators (TENGs) have attracted the most attention owing to their broad material compatibility, high efficiency, low weight, low fabrication cost, and inherent mechanical flexibility [7,8].

Triboelectric energy conversion occurs when two dissimilar materials are brought into contact and then separated, generating electricity via contact electrification and electrostatic induction. To design high-performance flexible TENGs, various strategies are utilized, such as chemical doping [9,10], device architecture [11] and surface modification [12,13]. Chemical doping alters dielectric properties of the active material for better charge transfer at the interface; efficient device architecture allows energy harvesting in multimodal contact electrification and surface modifications, and, in particular, can generate charge when two identical materials come into contact [14]. This raises the question of whether charge generation in a TENG arises solely from direct charge transfer between contacting surfaces, or whether additional mechanisms contribute to charge generation. For instance, hydrophobic surface modification in a water-droplet TENG utilizes kinetic energy in a multistep droplet falling process to facilitate electron transfer during contact electrification [15]. In solid-to-solid TENGs for increased charge generation, surfaces of active materials are modified to allow them to have high roughness. Mizzi et al. [16] proposed that flexoelectric polarization induced by strain gradients at the contact interface may significantly contribute to charge output, even in insulating polymers. The flexoelectric effect describes the coupling between a mechanical strain gradient and dielectric polarization: when two surfaces are brought into contact, local strain gradients generated at surface asperities can induce polarization in the underlying material. Varghese et al. subsequently demonstrated that a cellulose acetate/PDMS TENG incorporating pyramidal surface microstructures produces higher voltage and current than dome-patterned or flat analogs, attributing the improvement to enhanced flexoelectricity at the pyramid tips [17].

Despite these advances, quantitative models describing flexoelectric contributions in soft-material TENGs remain lacking. To address this gap, we fabricated an Ecoflex 00-10/NOA-63 (N/E) TENG with pyramid-patterned and truncated-pyramid-patterned contact surfaces using silicon lithography molds. NOA-63 was selected for its ease of demolding and mechanical rigidity, enabling faithful pattern transfer at the microscale. Ecoflex 00-10 serves as a soft counter-electrode that readily deforms under contact loading, thereby generating the strain gradients necessary for flexoelectric polarization. Critically, neither polymer exhibits intrinsic piezoelectricity, making the N/E TENG system an ideal platform for isolating and quantifying the flexoelectric contribution to charge output. We fabricated microscale pyramidal and truncated-pyramidal N/E TENGs, measured their charge output under controlled mass loading, and by combining soft-material indentation mechanics with existing hard-to-hard flexoelectric theory, developed a total charge equation that separates triboelectric and flexoelectric contributions. The resulting predictive model provides a practical tool for designing pyramid-structured TENGs with optimized charge output.

## 2. Materials and Methods

### 2.1. Silicon Mold Fabrication

**Figure 1:**
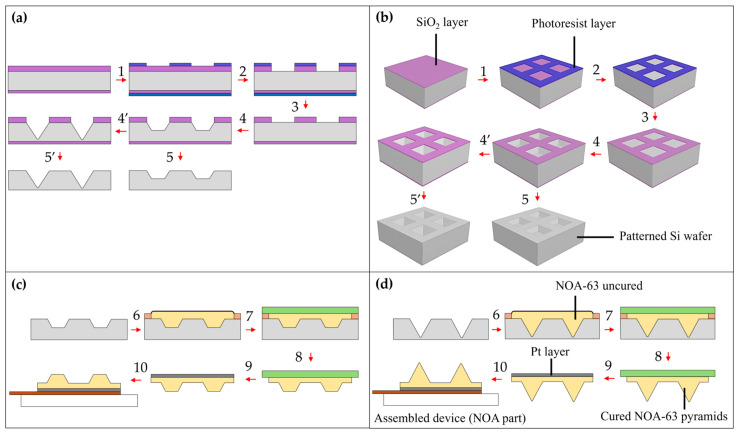
Schematic of silicon mold fabrication: (**a**) side-view and (**b**) 3D-view; NOA-63 electrode fabrication for (**c**) truncated-pyramidal and (**d**) pyramidal arrays.

The fabrication sequence for the silicon (Si) mold is illustrated schematically in Figure 1a,b, which are the side view and the 3-dimensional view of the fabrication process, respectively. In the beginning of the fabrication, a four-inch Si wafer coated with a 300 nm thermal silicon dioxide (SiO_2_) layer is shown. It was cleaned sequentially with acetone, ethanol, and deionized (DI) water under sonication (1 min each), dried under N_2_, and dehydrated on a hotplate at 95 °C for 1 min. The wafer surface was then primed with hexamethyldisilazane (HMDS; Samchun, Pyeongtaek, South Korea) by spin coating at 4000 rpm for 30 s. A 1.4 µm thick positive photoresist (PR; AZ5214-E, Merck, Darmstadt, Germany) was subsequently spin-coated at 4000 rpm for 30 s and soft-baked at 95 °C for 5 min.

Figure 1a,b start with the PR-coated wafer undergoing step 1 in which selective UV exposure through a chrome photomask (17.8 mW/cm^2^, 2 s) was followed by development in AZ300 MIF (Merck) for 1 min at room temperature. The patterned wafer was rinsed with DI water and dried under N_2_. In step 2, the exposed SiO_2_ was etched using buffered oxide etchant (BOE; Samchun) for 3 min at room temperature, with the PR layer serving as an etch mask. After stripping the PR via the developer shown in step 3, anisotropic wet etching of the Si in tetramethylammonium hydroxide (TMAH) solution at 90 °C (bath circulator CW3-20, Jeiotech, Daejeon, South Korea) is shown in steps 4 and 4’ of Figure 1a,b, which produced inverted pyramidal trenches. The shorter etching time in TMAH shown in step 4 leads to incomplete trench formation and thus a truncated-pyramidal mold is shaped, while complete etching yields a pyramidal mold, as shown in step 4’. Steps 5 and 5’ display the removal of the remaining SiO_2_ layer from the truncated pyramidal and pyramidal molds, respectively, for further usage in synthesis.

### 2.2. Device Fabrication

#### 2.2.1. NOA-63 Pyramid Layer

In Figure 1c,d, the step-by-step fabrication schemes of NOA-63 truncated pyramidal and pyramidal arrays are presented, respectively. Norland Optical Adhesive 63 (NOA-63, Norland Products, Jamesburg, NJ, USA) was poured onto the Si molds and degassed in a vacuum desiccator for 20 min to eliminate trapped air (step 6). In steps 7 and 8, the resin was pressed and UV-cured (5.4 mW/cm^2^, 20 min; MT-UV-A 17, Minuta Technology, Cheonan, South Korea), and then demolded to yield a freestanding pyramid-patterned polymer sheet. Step 9 shows that a thin platinum (Pt) electrode was deposited onto the flat backside of the NOA-63 sheets by ion sputtering (Q150R S, Quorum Technologies, Puslinch, ON, Canada).

#### 2.2.2. Ecoflex Substrate

Ecoflex 00-10 (Smooth-On, Macungie, PA, USA) base and curing agent were mixed at 1:1 mass ratio, degassed for 20 min, and spin-coated at 740 rpm for 200 μm and 400 rpm for a 400 μm thickness film for 30 s onto a temporary donor substrate (see Supplementary Material, Figure S1). The film was thermally cured at 70 °C for 2 h, released from the substrate, and coated with a Pt electrode by ion sputtering. Layer thickness was controlled by adjusting spin speed.

#### 2.2.3. Device Assembly

The NOA-63 pyramid sheet and Ecoflex substrate were assembled in a facing configuration with their insulating surfaces opposed, separated by compliant springs to maintain the contact–separation geometry. In step 10 of Figure 1, electrical connections were established by attaching copper tape to the Pt electrodes using silver epoxy.

### 2.3. Characterization

Surface morphology and feature dimensions pyramidal and truncated-pyramidal arrays were characterized by scanning electron microscopy (SEM, JCM-7000, JEOL, Tokyo, Japan). Charge output measurements were performed using a Keithley 6517B electrometer in contact–separation mode under applied mass loads ranging from 1 N to 40 N using a motorized force stage (JSV-H1000, JISC, Sakurai, Japan). A digital camera and ImageJ v1.54g software were used to capture and analyze images of deformation patterns of macroscale pyramidal and truncated pyramidal indenters.

## 3. Results and Discussion

### 3.1. SEM Images and Array Dimensions

**Figure 2:**
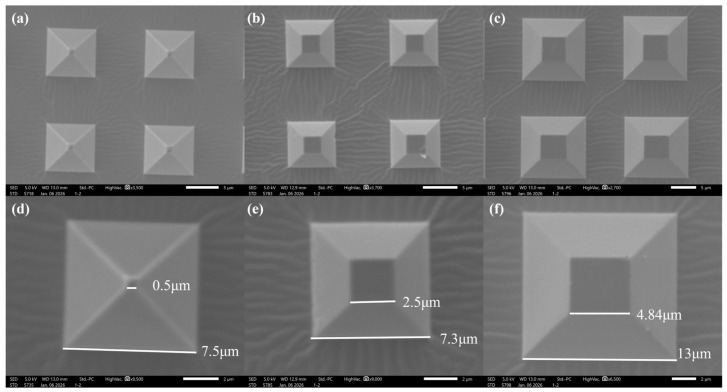
SEM images presenting three different NOA-63 pyramidal arrays confirming uniform structure and spacing. Top views of (**a**) array of 7.5 µm base-sized pyramid (P-7.5), (**b**) array of 7.5 µm base-sized truncated pyramid (TP-7.3) and (**c**) array of 13 µm base-sized truncated pyramid (TP-13) are shown. SEM images of singular pyramids provide dimensions of singular pyramids in each array what allows interpretation of triboelectric and flexoelectric components in TENG. Top views of singular (**d**) P-7.5, (**e**) TP-7.3 and (**f**) TP-13 pyramids with their base and peak sizes are observed.

To ensure that the model we are proposing is accurate, we need to confirm that NOA microstructures are reliable and distributed evenly. Figure 2 shows SEM images of the fabricated NOA-63 pyramidal and truncated-pyramidal arrays. In Figure 2a–c, the top views of three different pyramidal arrays are presented. These images prove uniformity in the size, structure and spacing of pyramids. So, we successfully fabricated arrays of three different configurations: pyramids with 7.5 μm base size (P-7.5) shown in Figure 2d, truncated pyramids with 7.3 µm base size (TP-7.3) shown in Figure 2e, and truncated pyramids with 13 µm base size (TP-13) shown in Figure 2f. Arrays of the corresponding structure have different pyramid peak sizes of 0.5 µm, 2.5 µm and 4.84 µm, respectively. It is important to keep in mind that in Figure 2c, the TP-13 array was made with a larger photomask and, therefore, has a lower number of pyramids per unit area.

### 3.2. Effect of Ecoflex Layer Thickness on Charge Output

In this study, independent consideration of triboelectricity and flexoelectricity for a thorough understanding of the contact electrification mechanism in an indentation is essential. To decouple triboelectric and flexoelectric contributions, it is first necessary to identify conditions under which the measured charge primarily reflects contact electrification, independent of changes in dielectric thickness. According to Song et al. [18], the surface charge density of a TENG depends on the dielectric capacitance, which varies inversely with dielectric thickness. In contact–separation mode, mechanical compression of a soft dielectric under applied load alters its thickness, introducing a systematic force-dependent artifact.

**Figure 3:**
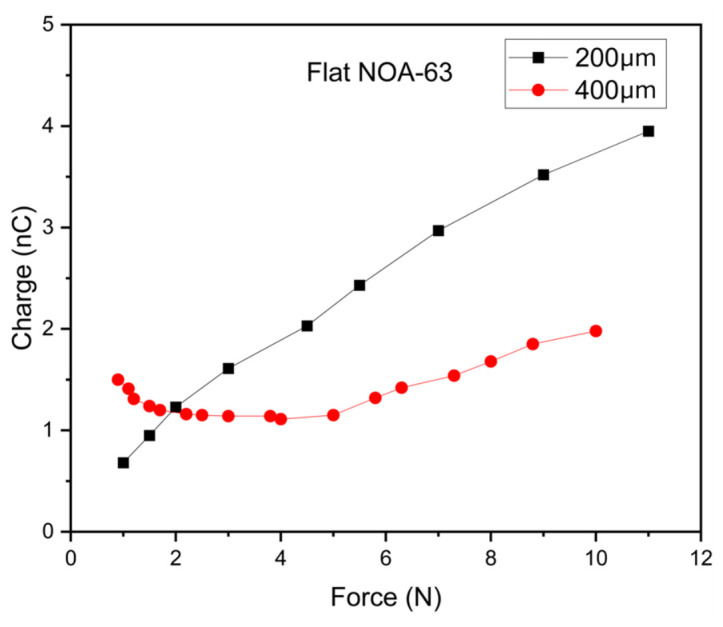
Charge output of flat NOA-63 paired with 200 µm (black) and 400 µm (red) Ecoflex films in contact–separation mode under applied loads from 1 N to 10 N. The 400 µm film shows load-independent behavior, confirming it as the optimal thickness for isolating triboelectric charge.

Typically, the thickness of polymers in a flexoelectric beam setup varies from 200 µm to 500 µm [19]. In the contact–separation setup, to avoid possible force-dependent artifacts, Ecoflex films of 200 µm and 400 µm thickness were paired with a flat NOA-63 counter-electrode and evaluated under mass loads from 1 N to 10 N. As shown in Figure 3, the 200 µm film exhibits a progressive increase in charge output with applied load, consistent with load-induced thickness reduction increasing capacitance. In contrast, the 400 µm film produces substantially more stable charge output across the load range, indicating that thickness-dependent effects are minimized. Accordingly, all subsequent measurements employed 400 µm Ecoflex films to ensure that charge output predominantly reflects contact electrification and flexoelectricity rather than thickness-variation artifacts.

### 3.3. Charge Output of Pyramidal and Truncated-Pyramidal Structures

**Figure 4:**
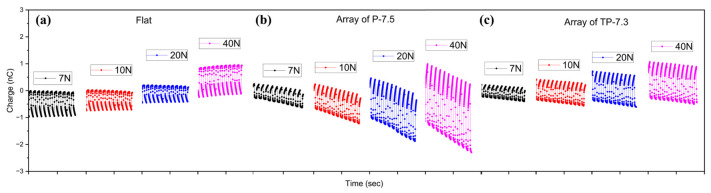
Representative charge-time traces for (**a**) flat, (**b**) array of pyramid with 7.5 μm base size (P-7.5), and (**c**) array of truncated pyramid with 7.3 μm base size (TP-7.3) N/E TENGs. As shown in subfigure (**b**), the pyramidal structure yields the highest contact-to-separation charge difference, consistent with enhanced flexoelectric polarization at the pyramid tip.

**Figure 5:**
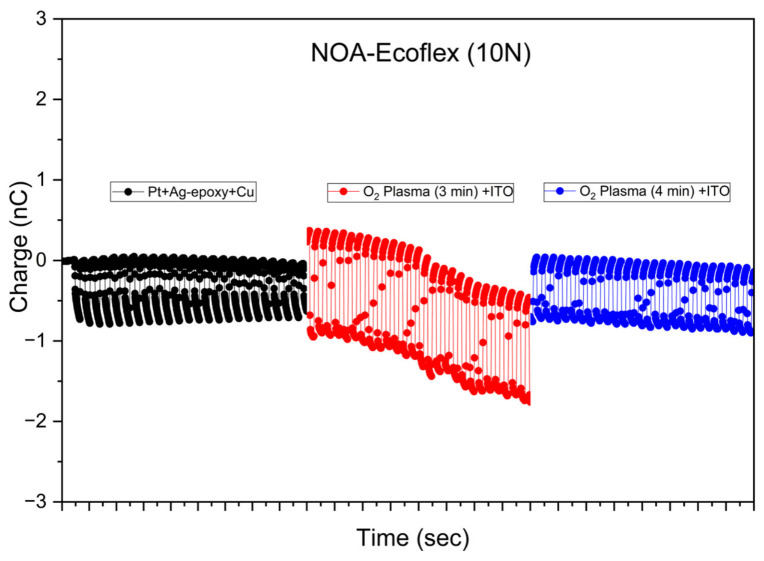
Charge output of non-surface-modified N/E TENGs devices assembled with rigid (Pt + Ag-epoxy + Cu) and flexible (O_2_ plasma + ITO) electrodes under 10 N load of Flat NOA sample.

Charge output of different samples was conducted after confirming NOA structures and adjusting Ecoflex thickness. Figure 4 presents representative charge-time traces for flat, P-7.5, and TP-7.3 N/E TENGs. A monotonic baseline drift was observed in all three samples during repeated cycling, while the contact-to-separation charge difference (ΔQ) remained largely stable. We are attributing drifting bias to capacitance originating from interface effects. Li et al. found that having gaps between the copper electrode and the triboelectric layer can enhance the capacitance of the device further [20]. In our N/E TENG device, the Pt layer is suffering from microcracks caused by large loads and patterned indenters. In comparison with current output from flat N/E TENG and TENG with TP-7.3 arrays, Figure 4b shows this feature the most because TENG with P-7.5 arrays creates localized sites where Ecoflex is compressed the most at the tips of pyramids, which translates into more severe cracks. The cracks on Pt layer can be seen in Figure S2 of the Supplementary Material, and those cracks create microscale gaps which cause interface capacitance effects [20]. To eliminate the capacitance effect in TENG devices, we assembled an N/E TENG with a flexible ITO/PET electrode. Since ITO has poor adhesion properties with Ecoflex, O_2_ plasma was used to improve ITO adhesion with Ecoflex. Figure 5 shows the charge output of N/E TENGs under a 10 N load with Pt/Ag-epoxy/Cu and O_2_ plasma-exposed ITO/PET electrodes. We found that 3 min plasma exposure was not enough to provide the necessary adhesion so that both charge drift was significant and in the middle of the test device, integrity was compromised. The longer plasma exposure time helped to ensure device stability, but baseline drift in current output was still in place. Even though having a flexible electrode solved the microcrack issue, poor adhesion with Ecoflex was still a major contributor to baseline drift. To eliminate drift-related bias, ΔQ values from the final 10 cycles of each measurement were averaged.

In Figure 4a, it is shown that flat NOA-63 generates the lowest charge, as it lacks geometric features that would induce significant strain gradients, and therefore its output is dominated by contact electrification. We also can observe from the figure that at 7 N force applied, the full contact between NOA and Ecoflex had been established, and at 10 N and 20 N, further compression of Ecoflex media leads to losing contact points due to the squeezing out of Ecoflex. However, at 40 N, we observe an increase in charge yield and, as it results in further Ecoflex compression, we link this change to the thickness effect described in the previous section. So, in further analysis, we limit the applied maximum force to 12 N to ensure proper contact of the active material and avoid the thickness effect.

Charge outputs contributed by both strain gradient-induced flexoelectricity and contact electrification are presented in Figure 4b,c. Both pyramidal arrays with less contact area yield higher charge output due to the strain gradient. The higher the difference in local pressure between the tip and base of a pyramid, the greater the strain gradient that will be formed. Therefore, the 7.5 µm pyramidal array exhibits the highest ΔQ value. Since pyramidal and truncated pyramidal arrays do not reach full contact at 7 N, we observe that at higher forces applied, both increased contact and thickness effects enhance charge output dramatically.

**Figure 6:**
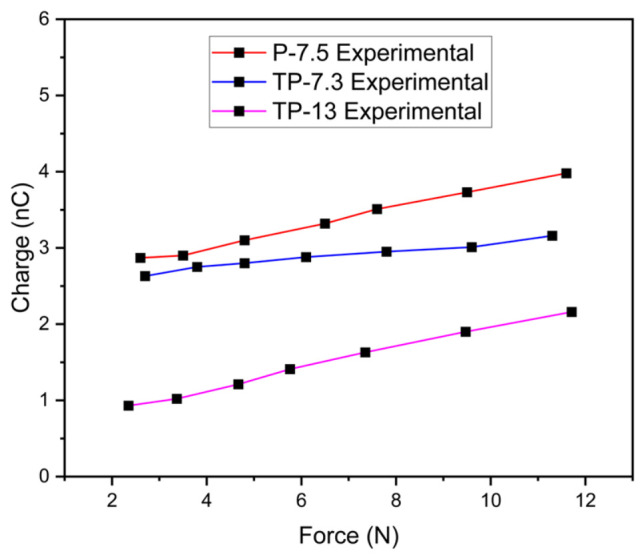
Mean ΔQ (averaged over final 10 cycles) versus applied force ranging from 2 N to 12 N for P-7.5, TP-7.3 and TP-13 N/E TENGs. The P-7.5 array with the highest flexoelectric contribution generates more ΔQ in contact–separation mode compared to the TP-7.3 and TP-13 arrays. The flexoelectric component is a major factor in ΔQ, and therefore the TP-13 array has the least charge output despite having the highest contact electrification among all configurations.

To obtain charge yields for our analysis, we again limited applied forces to ~12 N to avoid the thickness effect and extracted mean ΔQ values for the final 10 cycles of the P-7.5, TP-7.3, and TP-13 arrays. In Figure 6, it is shown that the P-7.5 array configuration consistently yields the largest ΔQ across all applied forces, attributable to the pronounced strain gradient at the pyramid apex, which drives flexoelectric polarization. The TP-7.3 and TP-13 arrays produce lower ΔQ, consistent with their reduced tip sharpness and correspondingly smaller strain gradients.

### 3.4. Contact Area and Triboelectric Contribution

**Figure 7:**
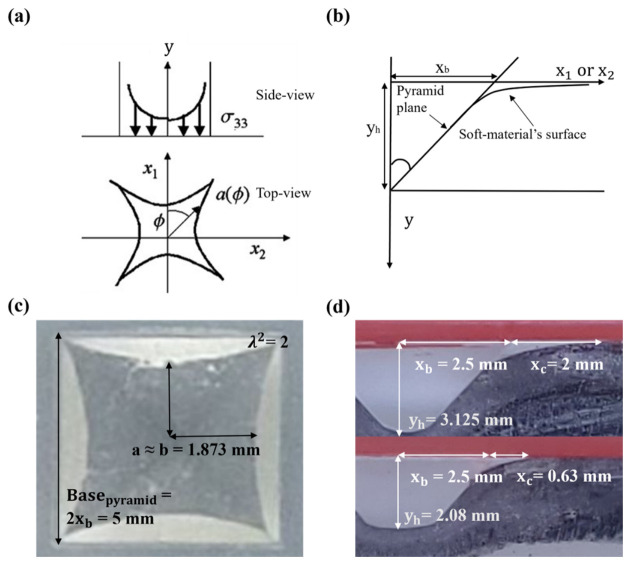
(**a**) Analytical contact perimeter profile with side and top views of flat punch with hyperbolic arc boundary and (**b**) theoretical cross-section surface deformation profile adapted from Giannakopoulos [14]. (**c**) Top-view photograph of a 5 mm macroscale NOA-63 pyramid indented into Ecoflex 00-10 with base half-distance (x_b_), hyperbola’s semi-diameters (a and b) and eccentricity (λ^2^). (**d**) Experimental cross-section profile of a 5 mm pyramid and a truncated pyramid with base half-distance (x_b_), contact distance (x_c_) and pyramid height (y_h_) used for local pressure and strain gradient calculations.

To quantify the triboelectric contribution to total charge, the contact area between the NOA-63 pyramids and the Ecoflex substrate must be established. Figure 7a,b describe the theoretical contact area while Figure 7c,d show microscope images of the real contact area of NOA indenter pushed into Ecoflex. Figure 7a represents a theoretical description of the flat punch with hyperbolic arcs and applied stress σ_33_ across the y-axis, and the top view of the indenter’s contact perimeter (a(*ϕ*)) within the x-axis at any given angle of contact radius (*ϕ*). Figure 7b shows the cross-section perspective of the indentation profile with a given half-distance of pyramid base (x_b_) and height of pyramid (y_h_). Comparing the theoretical model with experimental deformation data given in Figure 7c,d, we found that the contact profiles of the flat punch with hyperbolic arcs and the pyramidal indenter are identical, and thus the model can adequately quantify the contact area. Figure 7c shows the top view of the real deformation of the 5 mm pyramidal NOA indenter pressed into Ecoflex, from which we can extract the semi-diameter (b) and eccentricity (λ^2^) of the profile needed for Giannakopoulos’s contact perimeter equation (a(*ϕ*)) [21] shown in the Supplementary Material, (SM1). Figure 7d represents the real deformation patterns of pyramidal and truncated pyramidal indenters from a cross-section perspective. Their parameters, such as indentation depth across the y-axis (y_h_), half-distance of pyramid base (x_b_) and distance from pyramid base to contact point (x_c_), can be observed. The contact point (x_c_), in particular, is necessary to quantify the strain that is formed during the indentation. Data obtained from real indentation profiles provides the relation between pyramid base size and distance at which Ecoflex is in contact with the base plane, and this relation is applied to the microscale arrays we manufactured. When the macroscale deformation profile is being extrapolated into micro dimensions, the indentation size effect (ISE) must be considered. The ISE is significant in metals and in glass-like polymers and forbids such extrapolation due to a loss of macroscale homogeneity and microscale grain boundary interaction with a microscale indenter. Macroscale-to-microscale indentation transition leads to localized change in mechanical properties due to various grain boundaries and thus the deformation pattern may follow different scenarios [22]. Interestingly, polymers with less bulk monomer units such as polyethylene or polytetrafluorethylene have no change in hardness even at nanoscale indentation, while polystyrene and polycarbonate change their hardness at around 10 µm indentation [23]. This means that the mechanical properties of low-molecular-weight polymers are not changed in micro dimensions. Considering Ecoflex as a silicone variation with simple monomer blocks, macroscale-to-microscale extrapolation should have a minimal error.

With the hyperbola’s semi-diameter (b) and eccentricity (λ^2^), we can express contact area (A_pyramid_) as a function of the contact perimeter of the flat punch with hyperbolic arcs with a given indentation depth ‘h’ (y_h_) (Supplementary Material, SM1). By solving it, the resultant equation for contact area (A_pyramid_) can be simplified to $A_{Pyramid}=0.8892h^{2}$. Implementing this approach to the three array geometries yields total contact areas of $A_{Pyramid,7.5µm}\times N=3.48\times 10^{-5}m^{2}$, $A_{Pyramid,T-7.3µm}\times N=4.02\times 10^{-5}m^{2}$ and, $A_{Pyramid,T-13µm}\times N=7.37\times 10^{-5}m^{2}$. The triboelectric charge is then expressed as $Q_{tribo}=\sigma \times A_{pyramid}\times N$, where σ is the surface charge density. 

### 3.5. Strain Gradient and Flexoelectric Contribution

Flexoelectric polarization can be observed when any material is being bent and an inner arc of curvature is being compressed while the outer arc of curvature experiences tension leading to the formation of strain gradient within the thickness of the material. This phenomenon was expressed by Cross et al. [24]:

(1) $$P_{i}=\mu_{\mathrm{ijkl}}({dS}_{\mathrm{kl}}/{dx}_{j})$$

where P is the polarization, µ is the flexoelectric coefficient and dS/dx is the strain gradient. Adapting Equation (1) to our study, we can expand the equation into:

(2) $$P_{pyramid}=\mu_{flexo}\times \frac{dS}{dh}=\mu_{flexo}\times \frac{\frac{p_{tip}}{E_{Ecoflex}}-\frac{p_{bot}}{E_{Ecoflex}}}{∆h_{totaldepth}}$$

where E is Young’s modulus of the material, h is the total indentation depth, p_tip_ is the local pressure at the tip of the pyramid and p_base_ is the local pressure at the base of pyramid. The local pressure distribution within the Ecoflex film under a pyramidal indenter was described using Popov’s analytical solutions for linear conical profiles [25]:

(3) $$p\left(r,0{}^{\circ}\right)=\frac{E}{2}*1.125*\mathrm{arccosh}\left(\frac{a\left(0{}^{\circ}\right)}{r}\right)$$

where p is the local pressure, r is the radial distance from the pyramid center, and a(0°) is the contact radius at 0° azimuth. The appropriateness of Popov’s framework was confirmed by comparing predicted pressure contours shown in Figure S3b (see Supplementary Material, SM2) with experimental indentation profiles shown in Figure 7b, which show excellent agreement. Despite the fact that the theoretical model in Figure 7b has a horizontal asymptote at the y = 0, the real deformation pattern shows at what distance (x_c_) from the base Ecoflex is in contact with the NOA pyramidal indenter. So, the contact radius a(0°) can be expressed as a sum of x_b_ and x_c_ given in Figure 7d. When adapting local pressure from Equation (3) into the strain gradient of Equation (2), the contact depth can approach zero at low loads, so the total pyramid height was used as the reference length for strain gradient calculation to avoid unphysical divergence:

(4) $$P_{pyramid}=\mu_{flexo}\times \frac{dS}{dh}=\mu_{flexo}\times \frac{\frac{1.125}{2}\left[\mathrm{arccosh}\left(\frac{a\left(0\right)}{r_{tip}}\right)-\mathrm{arccosh}\left(\frac{a\left(0\right)}{r_{base}}\right)\right]}{∆h_{totaldepth}}$$

Strain gradients were determined by extrapolating macroscale indentation parameters presented in Figure 7c,d to the microscale dimensions measured from SEM images given in Figure 2d–f. The resulting values are: dS/dh_34_ = 3.35 × 10^5^ m^−1^ (P-7.5), dS/dh_34_ = 2.39 × 10^5^ m^−1^ (TP-7.3), and dS/dh_34_ = 1.30 × 10^5^ m^−1^ (TP-13). These values are physically consistent: sharper pyramid tips generate steeper strain gradients, and blunter (truncated) tips produce shallower gradients.

### 3.6. Total Charge Equation and Coefficient Extraction

Combining the triboelectric and flexoelectric contributions, the total charge generated by the N/E TENG is:

(5) $$Q=\sigma \times A_{pyramid}\times N+\mu_{flexo}\frac{\partial \epsilon }{\partial z}\times A_{sample}$$

where A_sample_ = 4 × 10^−4^ m^2^ is the total area of the Ecoflex sheet. The datasets of the TP-7.3 and TP-13 array N/E TENGs were selected to extract σ and μ_tlexo_ because their flat truncation tops are geometrically well-defined, reducing uncertainty in contact area estimation. At full indentation:

$$Q_{T-7.3\mu m}\;(at\;11.3N)=\sigma \times 4.02*10^{-5}m^{2}+\mu_{flexo}\times 2.39*10^{5}m^{-1}\times 4\times 10^{-4}m^{2}\;=3.16\times 10^{-9}C$$

$$Q_{T-13\mu m}\;(at\;11.71N)=\sigma \times 7.37*10^{-5}m^{2}+\mu_{flexo}\times 1.30*10^{5}m^{-1}\times 4\times 10^{-4}m^{2}\;=2.16\times 10^{-9}C$$

Solving this system of two linear equations yields σ = 8.48 × 10^−6^ C/m^2^ and μ_flexo_ = 2.95 × 10^−11^ C/m. Considering Ecoflex as a variation of PDMS with low molecular additives that interferes with cross-linking, the flexoelectric coefficients of Ecoflex μ_flexo_ = 2.95 × 10^−11^ C/m and PDMS μ_flexo_ = 5.8× 10^−11^ C/m [26] are similar with a correction to Young’s modulus and are consistent with the range of flexoelectric coefficients for polymers which is μ_flexo_ = 10^−11^~10^−10^ C/m [27].

### 3.7. Charge Estimation and Model Validation

**Figure 8:**
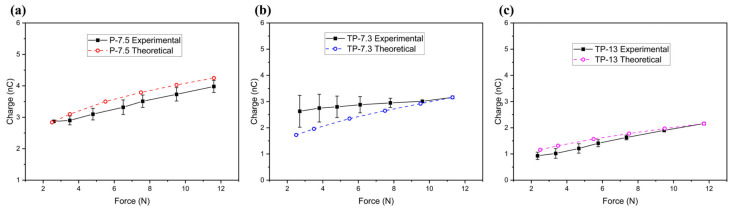
Output charge versus applied force of (**a**) P-7.5 array, (**b**) TP-7.3 array and (**c**) TP-13 array N/E TENGs. Solid symbols and hollow symbols with lines represent experimental data and theoretical predictions, respectively. Experimental data are compared with theoretical predictions estimated from Equation (5) using σ = 8.48 × 10^−6^ C/m^2^ and μ_flexo_ = 2.95 × 10^−11^ C/m.

The extracted coefficients were applied to estimate charge output as a function of applied force for all three array geometries, as shown in Figure 8. Indentation depth at each force level was determined by accounting for the spatial redistribution of load across the pyramid array. Using Popov’s normal force equation [25], an effective force of 5.11 N was found to act on the pyramids under a nominal applied load of 11.6 N for the P-7.5 array N/E TENG; the remaining force is balanced by the surrounding flat substrate. Effective forces at intermediate loads were computed similarly (Tables S1–S3 in the Supplementary Material) and used to determine force-dependent contact areas and strain gradients.

As shown in Figure 8a, the theoretical model for the P-7.5 array achieves the best agreement with the experiment, with a mean error of 7.26%, reflecting the well-characterized analytical behavior of sharp pyramidal indenters. As shown in Figure 8b,c, theoretical models for the TP-7.3 and TP-13 arrays demonstrate mean errors of 15.26% and 13.75%, respectively. Their truncation geometry was simplified in a way that the truncated contact area was modeled by subtracting the contribution of a smaller pyramid with base width equal to the truncation width, which may not fully capture the actual pressure distribution. This leads to a larger deviation for the TP-7.3 array displayed in Figure 8b. Interestingly, in Figure 8c, the TP-13 array also shows high concurrence with the theoretical model. Yet, we understand that, in this example, contact electrification is dominant and flexoelectricity is hindered due to the larger size of the pattern. This proves that the indentation profile is valid and describes the contact area accurately, while the flexoelectric driven deviation we observed in the TP-7.3 array is not prominent. To reach the highest possible accuracy for the prediction model of truncated pyramidal patterns, extensive study of soft-material deformation profiles under truncated pyramidal indenters is needed. This could be achieved by running simulation models and comparing results from the simulation with deformation patterns under truncated pyramid indentation. The challenge is to observe this process under a microscope and record it; the soft materials must be transparent with varying mechanical properties to prove the accuracy of the deformation pattern.

The total charge equation (Equation (5)) provides a reliable qualitative prediction of charge output across all three geometries and loading conditions, establishing it as a practical predictive tool for pyramid-structured TENG design, while from a quantitative perspective, this model is currently limited by an incomplete description of pressure distribution caused by the indentation profile of truncated pyramids. Future experimental studies characterizing soft-material pressure distribution under truncated-pyramidal indenters will enable the extension of this model to full quantitative predictive accuracy.

## 4. Conclusions

This study presents an analysis of flexoelectric contribution in hard-to-soft contact–separation TENGs with significant strain gradients. By fabricating NOA-63/Ecoflex TENG devices with precisely defined pyramid and truncated-pyramid microstructures and combining experimental charge measurements with analytical contact mechanics and flexoelectric theory, we could define a surface charge density of σ = 8.48 × 10^−6^ C/m^2^ and a flexoelectric coefficient of μ_flexo_ = 2.95 × 10^−11^ C/m for the N/E TENG system. The charge outputs of surface-modified samples at full indentation were found to be Q_P-7.5_ = 3.98 × 10^−9^ C, Q_TP-7.3_ = 3.16 × 10^−9^ C and Q_TP-13_ = 2.16 × 10^−9^ C. The N/E TENG with a 7.5 µm base size pyramidal array has the highest charge output due to its shape, which results in the highest strain gradient. In comparison to the charge output of the 7.5 µm pyramidal array N/E TENG, the charge output of the PDMS-based pyramidal array TENG was found to be Q_PDMS_ = 7 × 10^−9^ C when compressed under a 20 N load with similar array parameters [28]. However, the flexoelectric contribution to total charge output during the deformation of the surface-modified PDMS TENG remains unclear.

Defining flexoelectric and triboelectric components allows us to introduce the first quantitative model for predicting charge output in hard-to-soft contact–separation TENGs with significant flexoelectric contributions. The total charge equation (Equation (5)) successfully predicts the charge outputs of N/E TENGs with a 7.5 µm base size pyramid array and with 7.3 µm and 13 µm base size truncated pyramid arrays with mean errors below 8% for pyramidal and within acceptable bounds for truncated-pyramidal structures. The prediction model presented in this study can be improved with deep analysis of deformation patterns under truncated-pyramid indentation and by refining it with a wider selection of patterns. The framework established here is generalizable to other geometric configurations and material combinations, providing a rational basis for the design of next-generation flexible and wearable TENGs.

## Data Availability

The data presented in this study are available on request from the first author.

## Supplementary Materials

The following supporting information can be downloaded at: https://www.mdpi.com/article/10.3390/nano16140855/s1, Figure S1: (a) Ecoflex with 200 µm thickness and (b) 400 µm thickness film made at 740 rpm and 400 rpm for 30 s, respectively; Figure S2: Images of Pt layer deposited beneath Ecoflex before and after experiment, showing crack formation after applied load; Figure S3: (a) Pressure ratio versus normalized radial distance. (b) Boundary element method (BEM) simulation and (c) analytical solution for pressure distribution beneath a pyramidal indenter, confirming high local pressure at the apex and decreasing pressure toward the base [25]; Table S1. Theoretical charge estimation—pyramidal array with 7.5 µm base (P-7.5); Table S2. Theoretical Charge Estimation—Truncated-Pyramidal Array with 7.3 µm base (TP-7.3); Table S3. Theoretical Charge Estimation—Truncated-Pyramidal Array with 13 µm base (TP-13); SM1: Contact area derivation; SM2: Local pressure simplification; SM3: Strain gradient calculation; SM4: Effective force and charge estimation. References [29,30] are cited in the Supplementary Materials.
