# Supplementary material for: Flexoelectricity in Pyramid-Patterned Contact Areas of NOA/Ecoflex Triboelectric Nanogenerators
Source: Nanomaterials (Basel). 2026 Jul 11;16(14):855. doi: 10.3390/nano16140855 (PMC13415030; doi:10.3390/nano16140855)
Supplement: Supplementary file 1 [file nanomaterials-16-00855-s001.zip › nanomaterials-4376024-supplementary.pdf]

nanomaterials

Supplementary Materials

Flexoelectricity in Pyramid-Patterned Contact Areas of NOA/Ecoflex Triboelectric Nanogenerators

Nursalim Akhmetzhanov [1], Dong-Joo Kang [2], Jong-Man Kim [1,2], Dong-Myeong Shin [3] and Yoon-Hwae Hwang [1,2,*]

1 Department of Nano Fusion Technology & BK FOUR Nanoconvergence Technology Division, Pusan National University, Busan 46241, Republic of Korea; akhmetzhanov.nursalim@gmail.com (N.A.); jongkim@pusan.ac.kr (J.-M.K.)
2 School of Transdisciplinary Engineering, Pusan National University, Busan 46241, Republic of Korea; kdj9880@kakao.com (D.-J.K)
3 Department of Mechanical Engineering, The University of Hong Kong, Pokfulam Road, Hong Kong, China; dmshin@hku.hk (D.-M.S.)
* Correspondence: yhwang@pusan.ac.kr

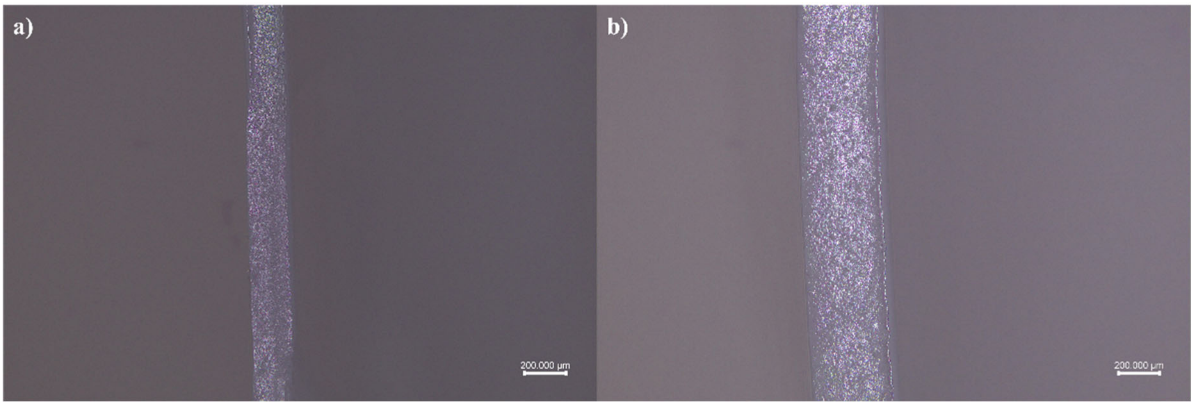

Figure S1. (a) Ecoflex with 200µm thickness and (b) 400µm thickness film made at 740 rpm and 400 rpm for 30 sec, respectively.

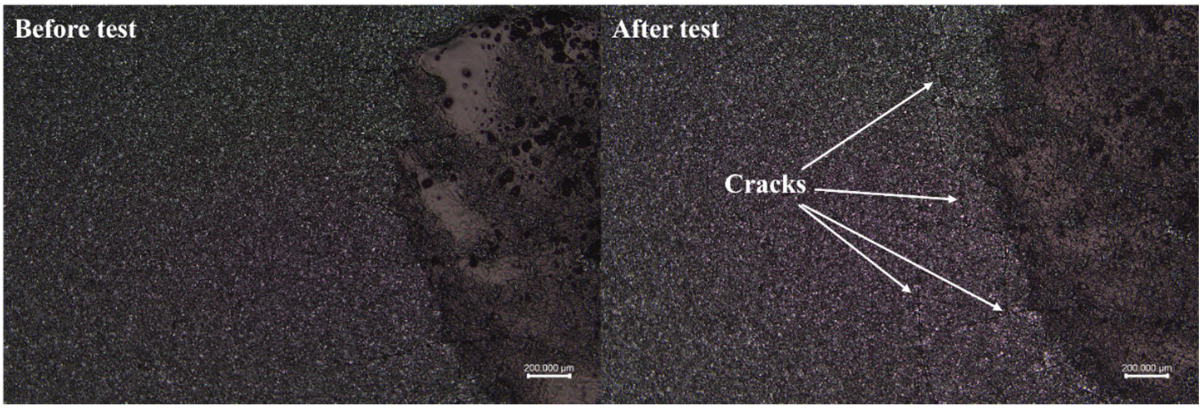

Figure S2. Images of Pt layer deposited beneath Ecoflex before and after experiment showing the cracks formation after applied load.

 https://doi.org/10.3390/xxxxx

SM1. Contact Area Derivation

Following Giannakopoulos [21], contact perimeter radius with given hyperbola parameters:

$$a(\phi) = \frac{b}{\sqrt{\lambda^2 cos^2\phi - 1}}$$

Pyramidal indenter's contact radius that has indentation depth can be expressed as contact area ($A_{pyramid}$):

$$A_{pyramid} = \frac{2\pi}{\Phi} * \frac{b^2}{2\sqrt{\lambda^2-1}}\left(\arctan\left(\frac{(\lambda+1)\tan\left(\frac{\Phi}{2}\right)}{\sqrt{\lambda^2-1}}\right) - \arctan\left(\frac{(\lambda-1)\tan\left(\frac{\Phi}{2}\right)}{\sqrt{\lambda^2-1}}\right)\right)$$

For a four-sided pyramid, $\Phi = \pi/4$, and with the parameters $\lambda = \sqrt{2}$ and b = 0.6h derived from macroscale indentation profiles (Figure 6c), equation reduces to $A_{pyramid} = 0.8892h^2$. For the 7.5 μm base size pyramidal (P-7.5) array (mask pitch 15 μm, sample area 4 cm^2): $N = 4 \times 10^{-4}$ m^2 / $(15 \times 10^{-6}$ m$)^2 = 1.78 \times 10^6$ pyramids; $A_{pyramid,7.5\mu m} = 0.8892 \times (4.69 \times 10^{-6}$ m$)^2 \times 1.78 \times 10^6 = 3.48 \times 10^{-5}$ m^2. For truncated pyramid arrays, the flat top area is added and the removed tip area subtracted accordingly (see main text for results section 3.4).

SM2. Local Pressure Simplification

Popov's analytical solutions for a linear conical indenter (k = 1, n = 4) provide the following set:

$$\bar{a} = \frac{2h}{\pi\bar{\psi}},\ F_N = \frac{2Eh^2}{\pi\bar{\psi}},\ a(\varphi) = \frac{2}{\pi}\frac{h}{\bar{\psi}}\left(\frac{5}{2} - \frac{3}{2}\frac{\psi(\varphi)}{\bar{\psi}}\right),\ p(r,\varphi) = \frac{E}{2}\bar{\psi} * \operatorname{arccosh}\left(\frac{a(\varphi)}{r}\right), \left(0 < r \le a(\varphi)\right).$$

From macroscale pyramid parameters ($h = 3.125 \times 10^{-3}$ m, $r = 2.5 \times 10^{-3}$ m), $\psi(0) = h/r = 1.25$ and $\bar{\psi} = (4/\pi)\psi(0)\sin(\pi/4) = 1.125$, giving $p(r, 0°) = (E/2) \times 1.125 \times \operatorname{arccosh}(a(0)/r)$ as presented in the main text (Equation 3). Figure S3 shows theoretical pressure distribution for pyramidal indenter. Boundary element method (BEM) simulations and analytical contours of pressure distribution given in Figures S3b and S3c are consistent with real deformation profile presented in Figure 7c which confirms validity of using local pressure equation (Equation 3) to interpret strain gradient in pyramidal indenter.

https://doi.org/10.3390/xxxxx

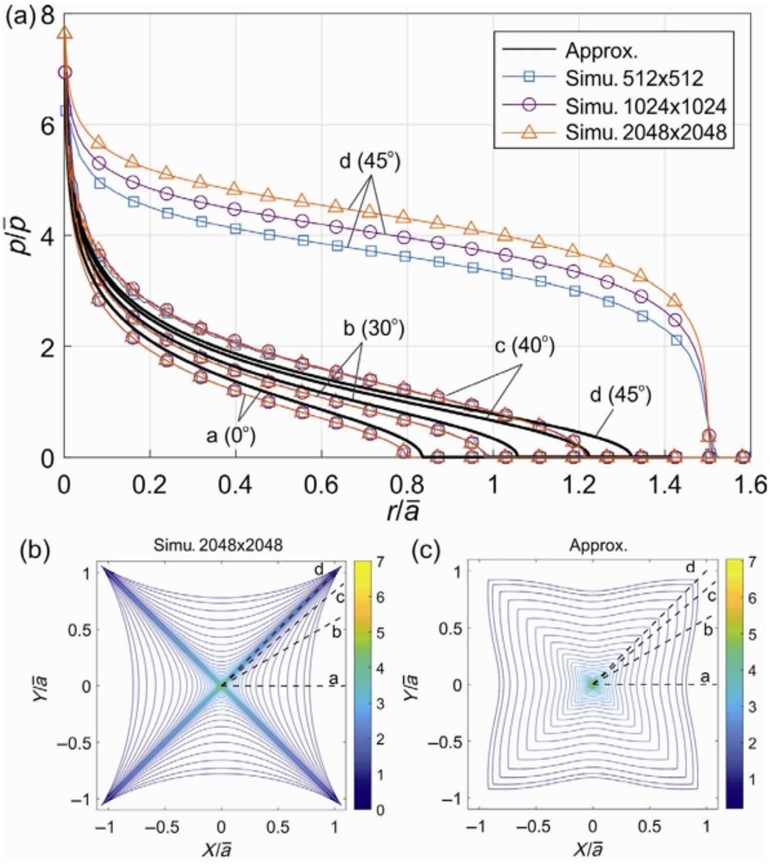

Figure S3. (a) Pressure ratio versus normalized radial distance. (b) Boundary element method (BEM) simulation and (c) analytical solution for pressure distribution beneath a pyramidal indenter, confirming high local pressure at the apex and decreasing pressure toward the base [25].

SM3. Strain Gradient Calculations

Part 1: 7.5 μm base size pyramid (P-7.5): Macroscale contact radius parameters (r_{base} = 2.5 × 10^{-3} m, a(0) = 4.5 × 10^{-3} m) were scaled to the microscale, giving r_{base} = 3.75 × 10^{-6} m, a(0) = 6.75 × 10^{-6} m, h = 4.69 × 10^{-6} m, and r_{tip} = 0.25 × 10^{-6} m. Substituting into Equation 4 yields dS/dh = 3.35 × 10^{5} m^{-1}.

Part 2: 7.3 μm (TP-7.3) and 13 μm (TP-13) base size truncated pyramids: For the truncated geometry, the effective contact radius at 0° was adjusted to $a(0)_{T\text{-}5mm}$ = 5 × 10^{-3} m/2 + 0.63 × 10^{-3} m = 3.13 × 10^{-3} m. After scaling: for TP-7.3, dS/dh = 2.39 × 10^{5} m^{-1}; for TP-13, dS/dh = 1.30 × 10^{5} m^{-1}. Full derivations are given in the main text, section 3.5.

SM4. Effective Force and Charge Estimation

The effective force per unit area applied to the pyramid array was computed using Popov's normal force equation:

$$F_{effective}(per\ Area) = \frac{2E^{*}d^{2}}{\pi\psi},\ \frac{1}{E^{*}} = \frac{1-\nu^{2}}{E_{Ecoflex\ 00-10}} + \frac{1-\nu^{2}}{E_{NOA-63}}$$

Using $E_{Ecoflex}$ = 43.3 kPa [1], $\nu_{Ecoflex}$ = 0.5, $E_{NOA\text{-}63}$ = 1743 MPa [2], and $\nu_{NOA\text{-}}^{623}$ = 0.35, the reduced modulus is E* = 5.77 × 10^{4} Pa. At full indentation of a 7.5 μm pyramid, $F_{effective,single}$ = 7.18 × 10^{-7} N, and the total effective force over the 4 ^{cm2} sample is 5.11 N, representing a redistribution ratio of approximately 2.27 relative to the applied 11.6 N load. Charge estimates at intermediate loads were obtained by solving for indentation depth from the

effective force, then computing contact area and strain gradient at that depth. Tables S1–S3 summarize all computed quantities.

Table S1. Theoretical Charge Estimation – Pyramidal Array with 7.5μm base (P-7.5).

Force Applied (N)	Force Effective (N)	Indentation Depth (μm)	r_{base} (μm)	Contact Area (m²)	Strain Gradient (m⁻¹)
2.5	1.10	2.175	1.74	0.75×10^{-5}	2.35×10^{5}
3.5	1.54	2.57	2.05	1.05×10^{-5}	2.55×10^{5}
5.5	2.42	3.23	2.58	1.65×10^{-5}	2.85×10^{5}
7.5	3.30	3.77	3.01	2.25×10^{-5}	3.05×10^{5}
9.5	4.185	4.24	3.39	2.85×10^{-5}	3.21×10^{5}
11.6	5.11	4.69	3.745	3.48×10^{-5}	3.35×10^{5}

Table S2. Theoretical Charge Estimation –Truncated-Pyramidal Array with 7.3μm base (TP-7.3).

Force Applied (N)	Force Effective (N)	Indentation Depth (μm)	r_{base} (μm)	Contact Area (m²)	Strain Gradient (m⁻¹)
2.5	0.49	1.41	2.38	2.12×10^{-5}	1.31×10^{5}
3.5	0.68	1.67	2.58	2.38×10^{-5}	1.49×10^{5}
5.5	1.07	2.09	2.92	2.84×10^{-5}	1.785×10^{5}
7.5	1.46	2.44	3.20	3.26×10^{-5}	2.015×10^{5}
9.5	1.85	2.75	3.44	3.67×10^{-5}	2.21×10^{5}
11.3	2.20	3.00	3.65	4.02×10^{-5}	2.39×10^{5}

Table S3. Theoretical Charge Estimation – Truncated-Pyramidal Array with 13μm base (TP-13).

Force Applied (N)	Force Effective (N)	Indentation Depth (μm)	r_{base} (μm)	Contact Area (m²)	Strain Gradient (m⁻¹)
2.5	0.43	2.36	4.28	4.10×10^{-5}	6.9×10^{4}
3.5	0.60	2.79	4.62	4.52×10^{-5}	7.9×10^{4}
5.5	0.94	3.49	5.18	5.29×10^{-5}	9.5×10^{4}
7.5	1.29	4.09	5.66	6.01×10^{-5}	1.08×10^{5}
9.5	1.63	4.59	6.06	6.66×10^{-5}	1.19×10^{5}
11.71	2.01	5.10	6.47	7.37×10^{-5}	1.30×10^{5}

References

1. Nishikawa, T.; Yamane, H.; Matsuhisa, N.; Miki, N. Stretchable Strain Sensor with Small but Sufficient Adhesion to Skin. *Sensors* **2023**, *23*, 1774, doi:10.3390/s23041774.
2. Turcitu, T.; Armstrong, C.J.K.; Lee-Yow, N.; Salame, M.; Le, A.V.; Fenech, M. Comparison of PDMS and NOA Microfluidic Chips: Deformation, Roughness, Hydrophilicity and Flow Performance. *Micromachines* **2023**, *14*, 2033, doi:10.3390/mi14112033.
